# Modifiable risk factors for dementia, and awareness of brain health behaviors: Results from the Five Lives Brain Health Ireland Survey (FLBHIS)

**DOI:** 10.3389/fpsyg.2022.1070259

**Published:** 2023-01-12

**Authors:** Tim Dukelow, Erin Grace Lawrence, Liron Jacobson, Philip Vassilev, Ivan Koychev, Kinan Muhammed, Sean P. Kennelly

**Affiliations:** ^1^Cork University Hospital (CUH), Cork, Ireland; ^2^Unit of Psychological Medicine, Centre for Psychiatry and Mental Health, Wolfson Institute of Population Health, Queen Mary University of London, London, United Kingdom; ^3^Five Lives SAS, Tours, France; ^4^Department of Psychiatry, University of Oxford, Oxford, United Kingdom; ^5^Nuffield Department of Clinical Neurosciences, Level 6, West Wing, John Radcliffe Hospital, University of Oxford, Oxford, United Kingdom; ^6^Tallaght University Hospital, Dublin, Ireland; ^7^Trinity College Dublin, Dublin, Ireland

**Keywords:** brain health, dementia, risk reduction, cognition, behavioral change

## Abstract

Up to 40% of dementias globally are attributable to modifiable risk factors. Many existing studies examining attitudes to brain health are limited by a failure to consider a range of pertinent risk factors and associated barriers to protective behaviors. In Ireland, self-reported knowledge of dementia is poor compared to other conditions. In this context, the current study aimed to explore exposure to and awareness of specific modifiable risk factors for dementia. We also aimed to investigate whether exposure to these risk factors is associated with demographic and socioeconomic factors. A cross-sectional survey was administered to 555 voluntary participants in February 2022. The survey captured the following information: (1) Sociodemographic factors; (2) Exposure to, as well as knowledge of modifiable risk factors for dementia, namely diet, social interaction, exercise, hypertension, sleep, depression, smoking, alcohol consumption, cognitive stimulation, hearing impairment, diabetes, air pollution, and head injury. The study population comprised 551 participants (50.3% male; 49.6% female). Mean age was 59.7 years. Modifiable risk factors for dementia were prevalent. Relative to females, male gender was significantly associated with multiple risk factors. Whilst 65.6% of participants believed that lifestyle improvements can decrease a person’s risk of developing dementia, only 31.4% believed that dementia could be prevented. Head injury (90.9%, *n* = 500), low mental stimulation (85.3%, *n* = 469), and alcohol consumption (77.8%, *n* = 428) were the three most commonly recognized risk factors. Awareness was significantly greater in both university groups (undergraduate and postgraduate) for multiple risk factors. Our findings demonstrate that the distribution of exposure to modifiable risk factors for dementia is unequal across gender and age groups, and that awareness levels vary across risk factors. These findings highlight that focus surrounding dementia prevention should shift toward individual risk profiling and should be tailored toward an individual’s specific needs.

## 1. Background

Dementia is an umbrella term encompassing several conditions, the hallmark of which is a progressive deterioration in cognition and function (McKhann et al., 2011). Dementia is common and, in many societies, underdiagnosed (Gauthier et al., 2021). Although the rate of dementia incidence has been declining in recent years in some western societies (Prince et al., 2016; Wolters et al., 2020), global dementia prevalence is increasing (Nichols and Vos, 2021). Dementia accounts for a significant proportion of health and social care expenditure and this is projected to increase. By 2050 it is estimated that dementia-attributable spending globally will reach 1.6 trillion dollars (Velandia et al., 2022). Much of the cost associated with dementia relates to informal care (Wimo et al., 2018).

Recent years have seen an increasing focus on dementia treatment and prevention. As of January 2022, there were 143 agents in 172 clinical trials for Alzheimer’s disease (AD) (Cummings et al., 2022). Citing an urgent need for treatments, the Food and Drug Administration (FDA) in June 2021 granted accelerated approval for aducanumab in the treatment of AD (Dunn et al., 2021) but concerns over its real-world utility remain (Canevelli et al., 2021; Padovani et al., 2022).

**FIGURE 1:**
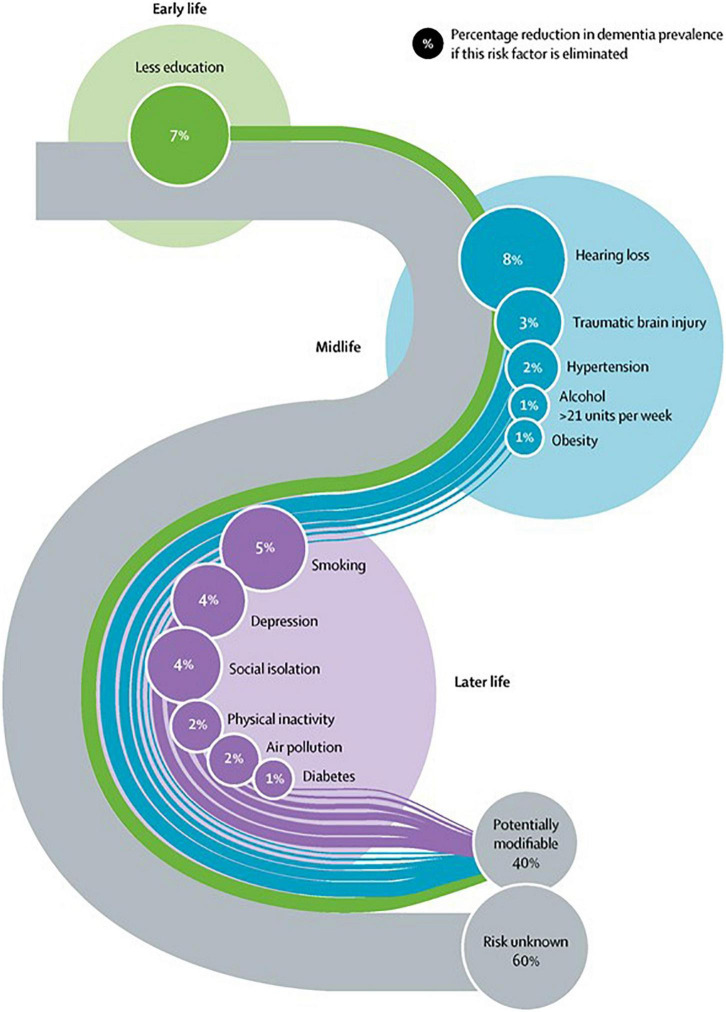
Reprinted from the Lancet, Vol. 396, Gill Livingston, Jonathan Huntley, Andrew Sommerlad, David Ames, Clive Ballard, Sube Banerjee, Carol Brayne, Alistair Burns, Jiska Cohen-Mansfield, Claudia Cooper, Sergi G. Costafreda, Amit Dias, Nick Fox, Laura N. Gitlin, Robert Howard, Helen C. Kales, Mika Kivimäki et al. Dementia prevention, intervention, and care: 2020 report of the Lancet Commission, Pages 413–446, Copyright (2020), with permission from Elsevier.

As disease modification options remain elusive, the concepts of brain health and dementia prevention through risk factor control have assumed a more prominent place in scientific literature and global health policy (WHO, 2017; Livingston et al., 2020). The use of the term brain health in medical literature has increased exponentially since 2011 (Chen et al., 2021). A 2021 concept analysis defined brain health as follows: “a life-long dynamic state of cognitive, emotional and motor domains underpinned by physiological processes. It is multidimensional and can be objectively measured and subjectively experienced. Brain health is influenced by eco-biopsychosocial determinants, resulting in a continuum of quality of life and wellness” (Chen et al., 2021). It is now recognized that up to 40% of dementias globally are attributable to modifiable risk factors (Livingston et al., 2020). On a related note, the observed decline in incidence rate of dementia in Europe and North America over the past 25 years, has been attributed to improved treatment options and outcomes for cardiovascular disorders (Wolters et al., 2020). The life-course model for modifiable dementia risk factors highlights that it is never too early or too late in the life course to consider dementia prevention (Figure 1). Whilst brain health services designed to operationalize primary and secondary dementia prevention measures are evolving, significant challenges remain in providing scalable programs and equity of access (Altomare et al., 2021).

Whilst a weight of evidence supports the potential for risk factor modification in reducing dementia risk (Livingston et al., 2020), health-promoting behaviors are not prevalent among the general population. Whilst modifiable risk factors for dementia disproportionately impact those in low-and middle-income countries, there is a relative paucity of research in these settings. Conversely, rates of physical inactivity are high in higher-income Western countries (Guthold et al., 2018) and adherence to cardiovascular medicines such as antihypertensives is poor (Naderi et al., 2012). Hearing aid use, a protective factor in the context of hearing impairment, varies considerably across studies (Smeeth et al., 2002; Lupsakko et al., 2005; Gimsing, 2008). Thus, while primary and secondary dementia prevention offers a tangible route to reduce its disease burden, the introduction of persistent behavioral change remains a challenge (Fineberg, 2013).

Misconceptions regarding the nature of dementia and its risk factors are prevalent and a barrier to the implementation of prevention programs. A recent systematic review of population-based surveys demonstrated that more than half of respondents in Europe and the United States believed that dementia was a part of normal ageing (Cations et al., 2018). Misconceptions regarding the pathologic nature of cognitive decline and the absence of treatment options have been recognized as contributing to the underdiagnosis of dementia (Bradford et al., 2009). Furthermore, a family history of dementia may skew perspectives and confer a sense of resignation to “one’s inevitable fate” (Kim et al., 2016). Relatedly, knowledge surrounding dementia risk reduction behaviors is often lacking (Smith et al., 2014). The importance of cardiovascular risk factors, for example, is poorly understood while risk reduction strategies of limited or no evidence are often endorsed by the general public (Cations et al., 2018).

Modifiable risk factors are unequally distributed in the general population due to their dependence on sociodemographic, cultural, and economic factors (Deckers et al., 2019; Röhr et al., 2022). Personalization of brain health interventions is thus critical and indeed individualized interventions are valued by patients (Curran et al., 2021). Such personalized preventative interventions are not a new phenomenon and have been provided in individual settings over the last decade (e.g., Seifan and Isaacson, 2015). A novel form of brain health service focused on delivering dementia risk profiling, risk communication, and risk reduction interventions has been proposed but equity of access, and indeed the widespread availability of such services, is not yet a reality (Milne et al., 2021). Better understanding of barriers to lifestyle change specific to dementia risk is crucial if large-scale, effective brain health interventions are to be developed.

Many existing studies examining attitudes to brain health are limited by a failure to consider a range of pertinent risk factors and associated barriers to protective behaviors. In Ireland, self-reported knowledge of dementia is poor compared to other conditions, and almost half of the population is unaware that dementia risk is modifiable (Hickey, 2019). In this context, the current study aimed to explore exposure to and awareness of specific modifiable risk factors for dementia. As previous research has reported an unequal distribution of modifiable risk factors in the general population (Bobrow et al., 2021; LaPlume et al., 2022), we also aimed to investigate whether exposure to these risk factors is associated with demographic and socioeconomic factors, and to explore whether modifiable risk factor awareness varies between groups.

## 2. Materials and methods

### 2.1. Study design and participants

The Five Lives Brain Health Ireland Survey (FLBHIS) is a cross-sectional survey that was distributed online among an Irish non-patient population. Participants were considered eligible for inclusion in this study if they were aged 50 years old and above, had no history of dementia diagnosis and had never worked in the healthcare sector. A pilot version of the survey was undertaken by 50 volunteers in January 2022. The survey was subsequently administered to a further 555 volunteers in February 2022. A Professional market research company, CDR Insights and Analytics Limited, was responsible for administration of the survey online to an existing market research panel. Consent was assumed when participants proceeded to complete and submit the survey.

Prior to commencement, this study was reviewed by The St. James’ Hospital/Tallaght University Hospital Joint Research Ethics Committee who advised that, in keeping with local institutional and legislative requirements, formal approval was not necessary.

### 2.2. Measures

The FLBHIS was developed through an iterative process. Informed by current literature and expert opinion, a 100-question survey was devised (see Supplementary Data Sheet 1). The survey was adapted from the Lifestyle Barriers for Cognitive Health Questionnaire (LBCHQ) (K. Muhammed 2021, personal communication, 2 September) to be suitable for an Irish population. Additional items relating to other modifiable risk factors for cognitive decline such as sleep issues (Spira et al., 2014) and mental stimulation (Krell-Roesch et al., 2019) were also added to the survey. The survey captured the following information: (1) Sociodemographic factors (gender, age, ethnicity, household income, level of education); (2) Exposure to, as well as knowledge of modifiable risk factors for dementia, namely diet, social interaction, exercise, hypertension, sleep, current low mood/depression, current smoking, alcohol consumption, cognitive stimulation, hearing impairment, diabetes, air pollution, and head injury. Awareness was measured on a Likert scale. Alcohol intake (> 14 units per week), being overweight, smoking, low physical activity, sleep quality, depression/low mood, low social interaction, low mental stimulation and residing in an area with high air pollution were self-reported risk factors, whereas diabetes, hypertension and hearing impairment were defined as being diagnosed by a healthcare professional. With regard to being overweight, subject perception was utilized as opposed to BMI to minimize any potential impact of missing data; (3) Barriers to brain health behaviors (reported separately); (4) Participants’ perceptions regarding potential for dementia prevention, and risk reduction (Likert scale).

### 2.3. Statistical analyses

All personal data was removed from the dataset prior to analyses. Analyses were conducted using StataMP 17.0 for Mac or IBM SPSS Statistics software for Windows (version 29). Frequency counts and percentages were used to show the sociodemographic characteristics and rates of exposure to modifiable risk factors for dementia among the sample. For all analyses, statistical significance was defined as *p* < 0.05. To investigate differences in awareness levels for modifiable risk factors across groups, 2-way mixed ANOVAs with Greenhouse-Geisser correction were performed. Three 2-way mixed ANOVAs were undertaken with gender, age, and education as respective between-subject factors. Each ANOVA had “risk factor” as a within-subject factor with 13 levels, each level representing one risk factor. The outcome measure for each ANOVA was awareness level measured by a Likert scale. Age was categorized into three groups, defined as 50–59 years old, 60–69 years old, and 70 years and above for the purpose of analysis. Due to small numbers in this group (*n* = 3), those who responded that they would prefer not to disclose their gender were excluded from the dataset for analyses. Education was categorized into three groups for the purpose of analysis, defined as primary/secondary school, university undergraduate, and university postgraduate levels. Due to small numbers who had been educated to primary school level (*n* = 10), this group was combined with those who had been educated to secondary school level. Adjustment for *post hoc* comparisons was undertaken using the Sidak correction. Binary logistic regression models were used to investigate the associations between exposure to modifiable risk factors for dementia, age, gender, and education presenting odds ratios (OR) and adjusted odds ratios (AOR) for the likelihood of reporting exposure to risk factors. Risk factor exposure variables that were measured on a Likert scale (social interaction, exposure to air pollution) were regenerated into binary variables for the purposes of analyses. Binary logistic regression analyses were adjusted for potential confounding factors such as household income and educational attainment, as well as for sex and age depending on the predictor variable used in the model.

## 3. Results

### 3.1. Sample characteristics

The study population ultimately comprised 551 participants. Of the 555 volunteers to whom the survey was originally administered, 3 participants were excluded as they did not disclose their gender. One further participant was excluded due to an incomplete dataset. Table 1 outlines the sociodemographic characteristics of the sample. The study population comprised 551 participants with an almost equal proportion of male and female respondents (50.3% male; 49.6% female). Mean age was 59.7 years, with the majority of the sample ranging between 50 and 59 years of age (54.3%). 98.9% of respondents were of White ethnicity. Most participants were educated to secondary school level or higher (98.2%), were employed or self-employed (52.2%), and cohabited with one or more persons (74.4%).

**TABLE 1:** Sociodemographic characteristics of the sample.

Characteristic	n (%)
**Age in years**	
50–64	437 (79.3)
65 and above	114 (20.7)
**Gender**	
Male	277 (50.3)
Female	274 (49.7)
**Educational attainment**	
Primary school	10 (1.8)
Secondary school	282 (51.3)
Undergraduate degree	196 (35.6)
Postgraduate degree	62 (11.3)
**Household income level**	
Less than €20,000	104 (18.9)
€20,000–€40,000	189 (34.4)
€40,000–€60,000	91 (16.6)
€60,000–€80,000	63 (11.5)
Above €80,000	48 (8.7)
**Employment status**	
Employed or self-employed	287 (52.2)
Unemployed	91 (16.6)
Retired	172 (31.3)
**Home circumstances**	
Living alone	141 (25.6)
Living with one other person	197 (35.8)
Living with more than one person	212 (38.6)

Total sample (*N* = 551). Not all totals for each variable sum to 551 due to missing data.

### 3.2. Exposure to modifiable risk factors

Exposure to modifiable risk factors for dementia across the sample is illustrated in Table 2. Modifiable risk factors for dementia were prevalent among the study population, where the most commonly reported exposures were being overweight (60.6%), having a lack of social engagement (54.9%), physical inactivity (42.7%), hypertension (36.7%), and self-assessed poor sleep quality (33.5%). The least common risk factor was exposure to activities with risk of head injury (1.8%). The impact of gender, age, and levels of education on exposure to modifiable risk factors are presented in Table 3 and summarized below.

**TABLE 2:** Exposure to modifiable risk factors.

Modifiable risk factor	n (%)
Overweight	333 (60.6)
Low social interaction	302 (54.9)
Low physical activity	215 (42.7)
Hypertension	202 (36.7)
Poor quality sleep	184 (33.5)
Alcohol consumption (14+ units/week)	119 (29.4)
Depression/Low mood	154 (28.0)
Smoking (> 1 cigarette/week)	125 (22.7)
Low mental stimulation	116 (21.1)
Air pollution	83 (15.1)
Hearing impairment	69 (12.6)
Diabetes (Type I or II, diet or medication controlled)	45 (8.2)
Activities with risk of head injury	10 (1.8)

Total sample (*N* = 551). Not all totals for each variable sum to 551 due to missing data.

**TABLE 3:** Association between exposure to modifiable risk factors age, gender, and education.

Risk factor	Predictors													
	**Age**			**Gender**				**Education**						
	**AOR change per 1 year increase**			**Male**			**Female**	**University undergraduate**			**University postgraduate**			**Secondary school**
	**AOR**	**95% Cl**	***P***	**AOR**	**95% Cl**	***P***	**AOR**	**AOR**	**95% Cl**	***P***	**AOR**	**95% Cl**	***P***	**AOR**
Blood pressure problems	**1.033**	**1.009–1.057**	**0**.**008**	1.252	0.876–1.788	0.217	1	0.94	0.643–1.375	0.75	0.678	0.373–1.233	0.203	1
Hearing loss	**1.066**	**1.032–1.100**	**<0**.**001**	1.303	0.764–2.222	0.331	1	0.748	0.422–1.324	0.319	0.573	0.227–1.444	0.237	1
Smoking	**0.94**	**0.911–0.971**	**< 0**.**001**	**1.618**	**1.065–2.457**	**0.024**	1	**0.468**	**0.296–0.738**	**0.001**	**0.199**	**0.076–0.520**	**<0**.**001**	1
Overweight	0.979	0.956–1.002	0.077	0.822	0.573–1.180	0.288	1	0.9	0.612–1.323	0.593	0.667	0.379–1.172	0.159	1
Lack of exercise	1.005	0.980–1.029	0.717	1.043	0.726–1.497	0.821	1	0.834	0.566–1.228	0.357	0.577	0.313–1.064	0.078	1
Depression	**0.959**	**0.932–0.986**	**0**.**004**	**0.678**	**0.462–0.996**	**0.048**	1	**0.611**	**0.401–0.930**	**0**.**022**	0.631	0.328–1.214	0.168	1
Diabetes	1.03	0.992–1.070	0.124	**2.141**	**1.097–4.176**	**0.026**	1	1.312	0.685–2.516	0.413	0.767	0.252–2.336	0.641	1
Low social interaction	**0.966**	**0.943–0.990**	**0**.**005**	0.777	0.539–1.122	0.178	1	1.213	0.818–1.800	0.337	1.18	0.652–2.136	0.584	1
Alcohol	0.993	0.964–1.023	0.623	**3.127**	**1.953–5.007**	**< 0.001**	1	0.933	0.577–1.507	0.776	0.509	0.220–1.178	0.115	1
Head injury	1.025	0.938–1.120	0.589	0.431	0.106–1.750	0.239	1	0.098	0.012–0.806	0.806	0.108	0.010–1.215	0.072	1
Air pollution	**0.956**	**0.923–0.991**	**0**.**013**	1.402	0.870–2.260	0.165	1	1.123	0.680–1.856	0.651	0.867	0.383–1.964	0.733	1
Sleep problems	**0.967**	**0.942–0.993**	**0**.**012**	**0.547**	**0.379–0.789**	**0.001**	1	0.847	0.571–1.256	0.409	0.789	0.429–1.454	0.448	1
Low mental stimulation	**0.945**	**0.915–0.976**	**<0**.**001**	**1.796**	**1.171–2.754**	**0.007**	1	**0.573**	**0.361–0.908**	**0.018**	**0.353**	**0.152–0.818**	**0**.**015**	1

Significant effects are reported in bold.

#### 3.2.1. Impact of gender on exposure to modifiable risk factors

Relative to females, male gender was significantly associated with an increased likelihood to report multiple risk factors, namely excess alcohol consumption (AOR 3.127, CI 1.953–5.007), smoking (AOR 1.618, CI 1.065–2.457), diabetes (AOR 2.141, CI 1.097–4.176), and low mental stimulation (AOR 1.796, CI 1.171–2.754). Males were also significantly less likely than females to report poor quality sleep (AOR 0.547, CI 0.379–0.789), and depression (AOR 0.678, CI 0.462–0.996).

#### 3.2.2. Impact of age on exposure to modifiable risk factors

Age differences are presented in Table 3 as AOR change per year increase in age. With increasing age, participants were less likely to report poor quality sleep (AOR 0.967, CI 0.942–0.993), low mental stimulation (AOR 0.945, CI 0.915–0.976), smoking (AOR 0.94, CI 0.911–0.971), depression (AOR 0.959, CI 0.932–0.986), low social interaction (AOR 0.966, CI 0.943–0.990), and exposure to air pollution (AOR 0.956, CI 0.923–0.991). Increasing age was also significantly associated with an increased likelihood of reporting diagnosis of hypertension (AOR 1.033, CI 1.009–1.057) and hearing impairment (AOR 1.066, CI 1.032–1.100).

#### 3.2.3. Impact of education on exposure to modifiable risk factors

Relative to the secondary school education group, participants educated to undergraduate and postgraduate level were significantly less likely to report smoking (AOR 0.468, CI 0.296–0.738; AOR 0.199, CI 0.076–0.520, respectively). The undergraduate group was less likely to report depression (AOR 0.611, CI 0.401–0.930). Relative to the secondary school education group, participants educated to undergraduate and postgraduate level were significantly less likely to report exposure to low mental stimulation (AOR 0.573, CI 0.361–0.908; AOR 0.353, CI 0.152–0.818).

### 3.3. Awareness of modifiable risk factors for dementia

65.6% of participants believed that lifestyle improvements can decrease a person’s risk of developing dementia. However, only 31.4% believed that dementia could be prevented with lifestyle modifications.

**FIGURE 2:**
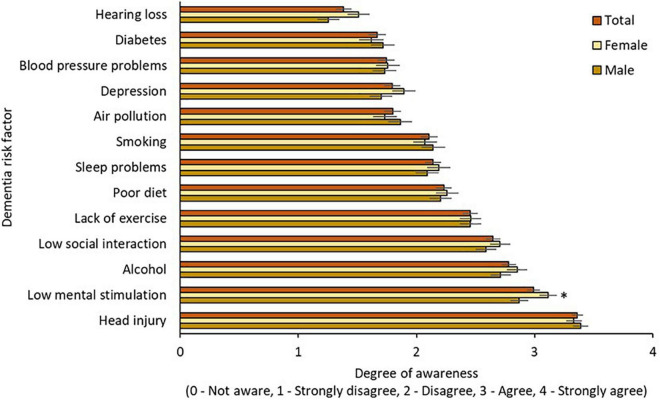
Awareness of modifiable risk factors by gender. * = sig. different from male group at 0.05 level.

**FIGURE 3:**
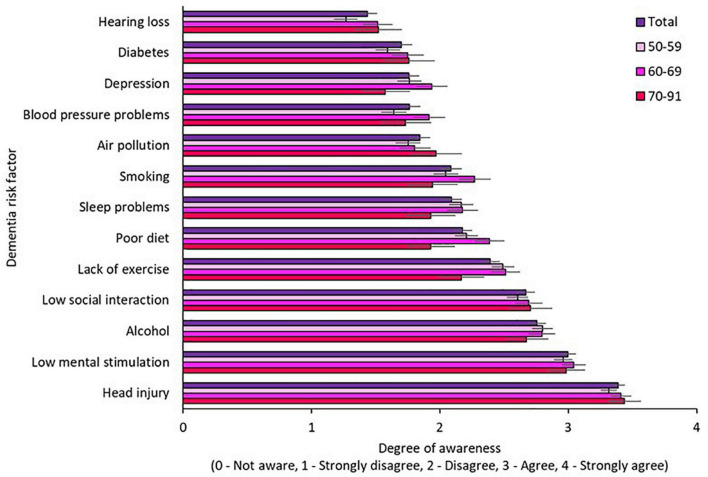
Awareness of modifiable risk factors by age.

**FIGURE 4:**
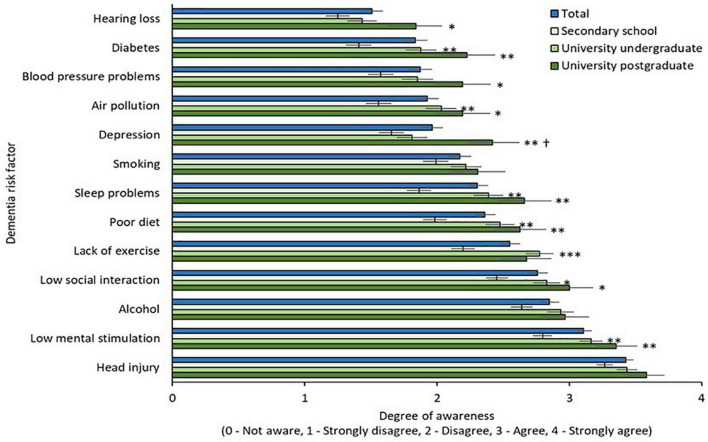
Awareness of modifiable risk factors by education. * = sig. different from secondary school group at 0.05 level; ^**^ = sig. different from secondary school group at 0.01 level; ^***^ = sig. different from secondary school group at 0.001 level; † = sig. different from university undergraduate group at 0.05 level.

Awareness of modifiable risk factors by age, gender, and education is illustrated in Figures 2–4, respectively. Across the total sample, head injury (90.9%, *n* = 500), low mental stimulation (85.3%, *n* = 469), and excess alcohol consumption (77.8%, *n* = 428) were the three most commonly recognized modifiable risk factors for dementia. Hearing impairment had the poorest recognition as a modifiable risk factor for dementia (34.7%, *n* = 191).

#### 3.3.1. Awareness of modifiable risk factors by gender

Awareness of modifiable risk factors by gender is illustrated in Figure 2. Two-way mixed ANOVA with Greenhouse-Geisser correction demonstrated significant main effects of risk factor [F(10.75,5888.82) = 134.88, *p* < 0.001] but not gender [F(1,548) = 0.48, *p* = 0.491] on awareness, and the interaction effect between risk factors and gender on awareness was not significant [F(10.75,5888.82) = 1.62, *p* = 0.09]. Awareness of low mental stimulation as a dementia risk factor was higher in the female group (*p* = 0.05).

#### 3.3.2. Awareness of modifiable risk factors by age

Awareness of modifiable risk factors by age is illustrated in Figure 3. Two-way mixed ANOVA with Greenhouse-Geisser correction revealed a significant main effect of risk factor [F(10.75,5879.15) = 93.71, *p* < 0.001] but not age [F(2.547) = 0.94, *p* = 0.393] and did not demonstrate a significant interaction effect between risk factors and age on awareness [F(21.49,5879.15) = 1.16, *p* = 0.279].

#### 3.3.3. Awareness of modifiable risk factors by education levels

Awareness of modifiable risk factors by education level is illustrated in Figure 4. Two-way mixed ANOVA with Greenhouse-Geisser correction revealed a significant main effect of risk factor [F(10.75,5881.99) = 82.69, *p* < 0.001] and main effect of education [F(2,547) = 11.99, *p* < 0.001]. The interaction effect between risk factors and educational level on awareness was not significant [F(21.51,5881.99) = 1.42, *p* = 0.09]. Awareness was significantly greater in both university groups (undergraduate and postgraduate) relative to the secondary education group for multiple risk factors, namely diabetes, air pollution, sleep, diet, low social interaction, and low mental stimulation (*p* = 0.01). Awareness of hearing loss, hypertension, and depression were significantly greater in the university postgraduate group when compared to the secondary school group (*p* = 0.05). Awareness of depression was greater in the postgraduate university group when compared to the undergraduate and secondary school groups (*p* = 0.05).

## 4. Discussion

In this study, we investigated the exposure to modifiable risk factors for dementia and their associations with gender, age, and education in an Irish sample of adults aged 50 and above. We assessed participants’ perception regarding the potential for dementia prevention, and risk reduction, as well as awareness levels of individual risk factors. Our findings demonstrate that the distribution of exposure to modifiable risk factors for dementia is unequal across gender, age, and education groups, and that awareness levels vary across risk factors, gender, and education level. These findings highlight that focus surrounding dementia prevention should shift toward individual risk profiling and should be tailored toward an individual’s specific needs.

Dementia has long been labeled a public health crisis (White, 1988). Dementia represents a public health priority for WHO member states, with cross-sectoral responses set out in the Global action plan on the public health response to dementia 2017–2025 (World Health Organization, 2017). The Global status report on the public health response to dementia highlights an urgent need to raise public awareness, and improve understanding of dementia, as well as the potential for risk reduction (World Health Organization, 2021). Our finding that a significant proportion of respondents felt that dementia cannot be prevented is in line with a global survey commissioned by Alzheimer’s Disease International (ADI) that demonstrated that two thirds of the general public, and 62% of healthcare practitioners believe that dementia is part of normal ageing (Alzheimer’s Disease International, 2019). Only 31.4% of people we surveyed believed that dementia could be prevented *via* lifestyle modifications. Despite this, 65.6% of participants believed that lifestyle improvements could decrease a person’s risk of developing dementia. This apparent disassociation between the concepts of prevention and risk reduction in the minds of participants perhaps speaks to a lack of reach or clarity in public health messaging on the subject. Ireland’s dementia awareness campaign, whilst impactful, has not been effective in increasing knowledge that dementia risk is modifiable (Hickey, 2019). Terminology is likely to be of particular importance in such contexts. It has been speculated that the term “risk reduction” may be a source of ambiguity with “prevention” representing a more tangible and actionable concept (Hodes et al., 2018).

In addition to a lack of awareness that dementia could be prevented *via* lifestyle modifications, knowledge of individual risk factors varied among the study group. High levels of awareness for certain risk factors may relate to the study population being relatively highly educated. 98.2% of survey respondents had attained at least secondary school level education as compared to 62% of older adults in a nationally representative Irish longitudinal study (Barrett et al., 2011). Our results are in line with a recent Dutch study of community dwelling older adults which found that individuals with lower levels of educational attainment were less likely to agree that dementia risk reduction is possible (Heger et al., 2019). In our study, participants educated to undergraduate university level or higher were significantly more aware of diabetes, air pollution, sleep, diet, low social interaction, and low mental stimulation as risk factors for dementia. Being educated to a post-graduate level was associated with additional awareness of the role of hearing loss, hypertension, and depression as compared to the secondary school group. This suggests a need to promote wider public awareness of these risk factors, especially considering that hearing loss is one of the most impactful mid-life risk factors for dementia with a population attributable fraction of 8% (Livingston et al., 2020). On the other hand, major depression is considered the fourth leading cause of morbidity globally according to the World Health Organization (WHO, 2008). Awareness of certain risk factors (air pollution, diabetes, hypertension, alcohol consumption) was broadly congruent with existing literature (Cations et al., 2018) and highlights the necessity to devise effective strategies to influence public perception. Dementia risk reduction campaigns are not a global phenomenon, with only 28 of 62 Global Dementia Observatory countries reporting having run such a campaign. The majority of campaigns were conducted in high income countries (World Health Organization, 2021). Only eight low-and middle-income countries report having run dementia risk reduction campaigns (World Health Organization, 2021), despite these nations being disproportionately impacted by modifiable risk factors and thus potentially having the greatest potential for dementia risk reduction (Livingston et al., 2020). Increasing dementia prevalence and the projected increase in costs associated with the disease make effective awareness campaigns encompassing dementia prevention a global imperative.

Modifiable risk factors for dementia were prevalent among study participants. More than half of those surveyed described themselves as being impacted by low social interaction, a well-established dementia risk factor (Penninkilampi et al., 2018). Conversely, high social engagement and large social networks are associated with better late-life cognitive function (Evans et al., 2019). Social engagement has been significantly impacted by the COVID-19 pandemic and its associated public health restrictions (Hwang et al., 2020) which raises the potential impact of this factor over the next decade. Loneliness and social isolation have been prevalent among older adults during the COVID-19 pandemic (Su et al., 2022) and mental and physical health have been negatively impacted over this period (Sepúlveda-Loyola et al., 2020). Fear of COVID-19 is prevalent amongst older adults (Agrawal et al., 2022). The promotion of social engagement as a means of dementia risk reduction is likely to prove challenging in this context.

Cardiovascular risk factors were prevalent amongst the study population (Table 2). Individual cardiovascular risk factors have been demonstrated to increase dementia risk (Livingston et al., 2020), and higher composite cardiovascular risk is associated with both vascular and AD dementia (Song et al., 2021). The role of brain vasculature in the pathogenesis of AD is increasingly recognized (Sweeney et al., 2018). Vascular risk factors have been demonstrated to potentially enhance neurodegeneration in individuals with preclinical AD (Bos et al., 2019). With regard to specific cardiovascular risk factors, 36.7% of study participants described a history of hypertension. Data from a large longitudinal Irish study suggests comparable hypertension prevalence but low levels of hypertension awareness, treatment, and control (Murphy et al., 2016). The number of adults with hypertension globally was predicted to increase by about 60% to a total of 1.56 billion by 2025 (Kearney et al., 2005). Whilst hypertensive awareness is improving (Mills et al., 2016), multiple studies have demonstrated poor adherence to antihypertensive medications (Vrijens et al., 2008; Algabbani and Algabbani, 2020). It has been speculated that lack of a “pay for performance” remuneration system may contribute to poor treatment and control in an Irish context (Murphy et al., 2016). We have demonstrated a substantial lack of awareness of hypertension as a risk factor for dementia (52.0% of those surveyed). It is plausible that this lack of knowledge may impact antihypertensive adherence.

Age profile and gender were independently associated with exposure to a variety of risk factors. Male gender, for example, was significantly associated with an increased likelihood to report multiple risk factors, namely excess alcohol consumption, smoking, diabetes, and low mental stimulation. Males were also significantly less likely than females to report poor quality sleep, and depression. This gender disparity, and spectrum of exposure across the age-range studied, highlights the need to avoid a “one size fits all” approach when it comes to dementia prevention. The concept of personalized primary and secondary prevention plans, tailored to an individual’s risk factor profile is central to proposed and evolving brain health services (Altomare et al., 2021).

This study is not without its limitations. This study comprises a population-based survey, distributed *via* a market research company to an existing online survey panel. This approach introduces the potential for selection bias. Furthermore, the degree to which these results are generalizable in a global context is unclear. Ireland is a wealthy, Western European nation with high levels of educational attainment (OECD, 2021). As highlighted, our study population appears highly educated with 98.2% having attained at least secondary school level education as compared to 62% of older adults in a nationally representative longitudinal study (Barrett et al., 2011). Online administration of the survey arguably represents a weakness as it further excludes individuals with low levels of education who may be lacking in technological literacy or do not have access to a digital device. Absence of laboratory investigations precluded computation of individualized dementia risk scores.

Strengths of the study include the detailed and robust survey design. The questionnaire comprised multiple questions, addressing both established and emerging risk factors. This study highlights ongoing knowledge deficits among the general public with regard to dementia risk factors and the potential for dementia prevention. Exposure to modifiable risk factors for dementia is considerable among this population of older adults. Individualized assessment and primary prevention in the context of specialist brain health clinics is unlikely to be impactful on a population level in the short to medium term. Novel health promotion and risk reduction approaches must be prioritized.

## Data availability statement

The raw data supporting the conclusions of this article will be made available by the authors, without undue reservation.

## Ethics statement

Ethical review and approval was not required for the study on human participants in accordance with the local legislation and institutional requirements. Written informed consent for participation was not required for this study in accordance with the national legislation and the institutional requirements.

## Author contributions

TD and SPK were responsible for the concept and study design, and also responsible for final review and editorial decisions. KM was responsible for design of the questionnaire on which the survey was based. TD, SPK, and EGL were responsible for amending and expanding the questionnaire. EGL and PV assisted with statistical analysis. TD and SPK undertaken the study writeup with input from LJ, EGL, PV, and IK. All authors contributed to the article and approved the submitted version.
