# Supplementary material for: Modifiable risk factors for dementia, and awareness of brain health behaviors: Results from the Five Lives Brain Health Ireland Survey (FLBHIS)
Source: Front Psychol. 2023 Jan 12;13:1070259. doi: 10.3389/fpsyg.2022.1070259 (PMC9879702; doi:10.3389/fpsyg.2022.1070259)
Supplement: Supplementary file 1 [file Data_Sheet_1.PDF]

Page 1

[NUMERIC] Q1: What is your age (in years)?

- [1]

[DROPDOWN] Q2: What is your gender

- [1] Male
- [2] Female
- [3] Prefer not to say

Page 2 - Logic : Q1 : 1 Age less Than 50

RUN ACTIONS ONLY IF **Q1_1 < 50**

Terminate respondent using code **Q1term2**

Page 3 - Logic : Q2 equals 3 - Gender not stated

RUN ACTIONS ONLY IF **Q2 = 3**

Terminate respondent using code **Gender not given**

Page 4

[DROPDOWN] Q4: What is the highest level of formal education you have completed?

- [1] Primary school
- [2] Secondary school
- [3] Undergraduate university degree
- [4] Post graduate university degree

[DROPDOWN] Q3: What is your current employment status?

- [1] Employed or self employed
- [2] Unemployed
- [3] Retired

Page 5

[RADIO] Q50: In which of the following sectors do you work?

Settings:

Skip logic: **(Q3 == 2 OR Q3 == 3)**

- [1] Education
- [2] Financial services
- [3] Government and public service
- [4] Law
- [5] Manufacturing
- [6] Healthcare
- [7] Retail
- [8] Other sectors

Page 6 - Logic : Q50 equals 6 Healthcare

RUN ACTIONS ONLY IF **Q50 = 6**

Terminate respondent using code **Q50term1**

Page 7

[RADIO] Q5: What is your weight (in kgs or stones)?

Instructions: Please select <u>only one type of answer</u>

- [1] In kgs [SPECIFY]
- [2] In stones [SPECIFY]
- [3] Don't know / Prefer not to say

[NUMERIC] Q6: What is your height (in cm or feet)?

Instructions: Please select <u>only one type of answer</u>

- [1] In cm (for example type: 160)
- [2] In feet (for example type: 5.6)
- [3] Don't know / Prefer not to say [DONTKNOW]

Page 8

[RADIO] Q7: How would you describe your ethnicity?

- [1] White
- [2] Mixed or multiple ethnic groups
- [3] Asian
- [4] Black, African, or Carribean
- [5] Irish traveller
- [6] Prefer not to say

Page 9

[RADIO] Q8: What is your annual household income level?

- [1] Less than €20 000
- [2] €20 000 - €40 000
- [3] €40 000 - €60 000
- [4] €60 000 - €80 000
- [5] Above €80 000
- [6] Don't know / Prefer not to say

[DROPDOWN] Q9: What best describes your home circumstances?

- [1] Live alone
- [2] Live with only one person (partner, child, family or friend)
- [3] Live with more than one person (children, family or friends)

Page 10 : Comparison to other diseases

[CHECKBOX] Q61: In this section, we would like to understand your view about **future health risks for you**

Settings:

Instructions: Which of these diseases are you most concerned about developing in the future? Tick all that apply

- [1] Cancer
- [2] Stroke
- [3] Dementia
- [4] Heart disease
- [5] Other
- [6] None in particular [DONTKNOW]

Page 11

[CHECKBOX] Q49: Which of these diseases do **you believe can be prevented with lifestyle modifications** (doing more exercise, eating healthy foods, quitting smoking, etc)?

Settings:

Min Count: **1**

Instructions: Tick the ones you believe can be prevented with lifestyle modifications, or select Don't know

- [1] Cancer
- [2] Stroke
- [3] Heart Disease
- [4] Dementia
- [5] Don't know [DONTKNOW]

Page 12

[RADIO] Q10: Have you seen a healthcare professional about memory worries?

- [1] YES
- [2] NO

Page 13

[CHECKBOX] Q11: Which of the following healthcare professionals have you seen for memory worries? Please tick all that apply

Settings:

Skip logic: **(Q10 == 2)**

- [1] General Practitioner only [DONTKNOW]
- [2] Neurologist
- [3] Psychiatrist
- [4] Geriatrician

[CHECKBOX] Q12: Have you been given a diagnosis by a healthcare professional of any of the following? Please tick all that apply

Settings:

Show logic: **(Q10 HAS [1])**

Min Count: **1**

- [1] Subjective Cognitive Impairment
- [2] Mild Cognitive Impairment
- [3] Dementia
- [4] Depression
- [5] Anxiety
- [6] None of these [DONTKNOW]

Page 14 - Logic : Q12 equals 3

RUN ACTIONS ONLY IF **Q12_3 = 1**

Terminate respondent using code **Dementia diagnosed**

Page 15

[SIMPLE_GRID][RADIO] Q62: Please indicate your level of agreement with this statement

Settings:

Mobile smart: **Yes**

Rows:

- [1] Lifestyle improvements can decrease a person's risk of developing dementia

Columns:

- [1] Strongly agree
- [2] Agree
- [3] Disagree
- [4] Strongly disagree
- [5] Don't know

Page 16

[SIMPLE_GRID][RADIO] Q13: In this section, we want to understand your attitudes regarding memory loss and dementia

Settings:

Mobile smart: **Yes**

Instructions: Please rate your level of agreement with each statement

Rows:

- [1] If necessary, I could make significant and sustained changes to my lifestyle
- [2] I am currently worried about my memory
- [3] I am worried I might have dementia

Columns:

- [2] Strongly agree
- [3] Agree
- [4] Disagree
- [5] Strongly disagree

Page 17 - Logic : Logic blocks straight line

LOOP ALWAYS

Initialise vars to zero to start

For each response in **SL_Q13** as X:

Page 18

[RADIO] Q18: I have been told by a healthcare professional that I have high blood pressure

- [1] YES
- [2] NO

Page 19

[SIMPLE_GRID][RADIO] Q20: Please indicate your level of agreement with this statement

Settings:

Mobile smart: **Yes**

Rows:

- [1] Having well controlled blood pressure is important in terms of reducing a person's future risk of dementia

Columns:

- [1] Strongly agree
- [2] Agree
- [3] Disagree
- [4] Strongly disagree
- [5] Don't know

Page 20

[SIMPLE_GRID][RADIO] Q19: In this section, we want to understand what are the barriers for you to maintain an optimal blood pressure

Settings:

Skip logic: **Q18 = 2**

Mobile smart: **Yes**

Instructions: Please rate your level of agreement with each statement

Rows:

- [1] If I had to make lifestyle changes to improve my blood pressure, I would know how to do this
- [2] If I had to make lifestyle changes to improve my blood pressure, I would be able to get healthcare support to do so

Columns:

- [1] Strongly agree
- [2] Agree
- [3] Disagree
- [4] Strongly disagree

Page 21

[SIMPLE_GRID][RADIO] Q19_CLONE: In this section, we want to understand what are the barriers for you to maintain an optimal blood pressure

Settings:

Skip logic: **Q18 = 2**

Mobile smart: **Yes**

Instructions: Please rate your level of agreement with this statement

Rows:

- [3] I would not get my blood pressure checked as I would be too worried about the outcome

Columns:

- [1] Strongly agree

- [2] Agree
- [3] Disagree
- [4] Strongly disagree

Page 22 - Logic : Logic blocks straight line

LOOP ALWAYS

Initialise vars to zero to start

For each response in **SL_Q19** as X:

Page 23

[RADIO] Q23: I have been told by a healthcare professional that I have a hearing impairment

- [1] YES
- [2] NO

Page 24

[SIMPLE_GRID][RADIO] Q57: Please indicate your level of agreement with this statement

Settings:

Mobile smart: **Yes**

Rows:

- [1] Correcting hearing difficulties may reduce the risk of future memory decline and dementia

Columns:

- [1] Strongly agree
- [2] Agree
- [3] Disagree
- [4] Strongly disagree
- [5] Don't know

Page 25

[SIMPLE_GRID][RADIO] Q24: In this section, we want to understand your attitudes regarding the treatment of potential hearing problems

Settings:

Skip logic: **Q23 = 2**

Mobile smart: **Yes**

Instructions: Please rate your level of agreement with each statement

Rows:

- [1] If I wanted to have my hearing assessed, I would know how to arrange this
- [2] If I had hearing difficulties, I would be too busy to arrange a hearing assessment

Columns:

- [1] Strongly agree
- [2] Agree
- [3] Disagree
- [4] Strongly disagree

Page 26

[SIMPLE_GRID][RADIO] Q24_CLONE_CLONE: In this section, we want to understand your attitudes regarding the treatment of potential hearing problems

Settings:

Skip logic: **Q23 = 2**

Mobile smart: **Yes**

Instructions: Please rate your level of agreement with this statement

Rows:

- [3] If I had hearing difficulties, I would not undertake a hearing assessment as I would be too worried about the outcome

Columns:

- [1] Strongly agree

- [2] Agree
- [3] Disagree
- [4] Strongly disagree

Page 27

[SIMPLE_GRID][RADIO] Q24_CLONE

Settings:

Skip logic: **Q23 = 2**

Mobile smart: **Yes**

Instructions: Please rate your level of agreement with each statement

Rows:

- [1] If I required a hearing aid, the cost (approximately €2 000) would put me off
- [2] If I required a hearing aid, I would be worried what people would think of me

Columns:

- [1] Strongly agree
- [2] Agree
- [3] Disagree
- [4] Strongly disagree

Page 28 - Logic : Logic blocks straight line

LOOP ALWAYS

Initialise vars to zero to start

For each response in **SL_Q24** as X:

Page 29

[RADIO] Q25: I smoke at least 1 cigarette per week

- [1] YES
- [2] NO

Page 30

[SIMPLE_GRID][RADIO] Q26: Please indicate your level of agreement with this statement

Settings:

Skip logic: **0**

Mobile smart: **Yes**

Rows:

- [1] Quitting smoking may reduce the risk of future memory decline and dementia

Columns:

- [1] Strongly agree
- [2] Agree
- [3] Disagree
- [4] Strongly disagree
- [5] Don't know

Page 31

[SIMPLE_GRID][RADIO] Q27: In this section, we want to understand your attitudes to smoking

Settings:

Skip logic: **Q25 = 2**

Show logic: **0**

Mobile smart: **Yes**

Instructions: Please rate your level of agreement with each statement

Rows:

- [1] I want to stop smoking but I haven't got the willpower to stop on my own
- [2] I don't think I could stop smoking when other people I live with continue to smoke

Columns:

- [1] Strongly agree
- [2] Agree
- [3] Disagree
- [4] Strongly disagree

Page 32

[SIMPLE_GRID][RADIO] Q27_CLONE: In this section, we want to understand your attitudes to smoking

Settings:

Skip logic: **Q25 = 2**

Show logic: **0**

Mobile smart: **Yes**

Instructions: Please rate your level of agreement with each statement

Rows:

- [3] I don't think I could stop smoking due to the social life I have
- [4] Stress and pressures from work or personal life mean I cannot quit smoking

Columns:

- [1] Strongly agree
- [2] Agree
- [3] Disagree
- [4] Strongly disagree

Page 33 - Logic : Logic blocks straight line

LOOP ALWAYS

Initialise vars to zero to start

For each response in **SL_Q27** as X:

Page 34

[RADIO] Q28: Do you think you are overweight?

- [1] YES
- [2] NO
- [3] Prefer not to say / Don't know

Page 35

[SIMPLE_GRID][RADIO] Q58: Please indicate your level of agreement with this statement

Settings:

Mobile smart: **Yes**

Rows:

- [1] A healthy diet may reduce the risk of future memory decline and dementia

Columns:

- [1] Strongly agree
- [2] Agree
- [3] Disagree
- [4] Strongly disagree
- [5] Don't know

Page 36

[SIMPLE_GRID][RADIO] Q29: In this section, we want to understand potential barriers for you to have a healthy diet

Settings:

Skip logic: **Q28 = 2**

Mobile smart: **Yes**

Instructions: Please rate your level of agreement with each statement

Rows:

- [1] I want to have a healthier diet but I do not have the motivation to do so
- [2] I want to have a healthier diet but I am unsure of what a healthy diet consists of

Columns:

- [1] Strongly agree
- [2] Agree
- [3] Disagree
- [4] Strongly disagree

Page 37

[SIMPLE_GRID][RADIO] Q29_CLONE: In this section, we want to understand what are the barriers for you to have a healthy diet

Settings:

Skip logic: **Q28 = 2**

Mobile smart: **Yes**

Instructions: Please rate your level of agreement with each statement

Rows:

- [3] The taste of healthy foods stops me from eating them regularly
- [4] Healthy foods do not satisfy my appetite and this stops me from eating them regularly

Columns:

- [1] Strongly agree
- [2] Agree
- [3] Disagree
- [4] Strongly disagree

Page 38

[SIMPLE_GRID][RADIO] Q56_CLONE

Settings:

Skip logic: **Q28 = 2**

Mobile smart: **Yes**

Instructions: Please rate your level of agreement with each statement

Rows:

- [3] People I live with do not enjoy healthy foods and this stops me from eating them regularly
- [4] Stress and pressures from my work or personal life cause me to eat less healthy meals

Columns:

- [1] Strongly agree
- [2] Agree
- [3] Disagree
- [4] Strongly disagree

Page 39

[SIMPLE_GRID][RADIO] Q56

Settings:

Skip logic: **Q28 = 2**

Mobile smart: **Yes**

Instructions: Please rate your level of agreement with each statement

Rows:

- [1] Healthy meals cost too much for me to eat them regularly
- [2] Healthy meals take too long to prepare for me to eat them regularly

Columns:

- [1] Strongly agree
- [2] Agree
- [3] Disagree
- [4] Strongly disagree

Page 40 - Logic : Logic blocks straight line

LOOP ALWAYS

For each response in **SL_Q29_CLONE** as X:

Page 41 - Logic : Logic blocks straight line

LOOP ALWAYS

Initialise vars to zero to start

For each response in **SL_Q29** as X:

Page 42

[RADIO] Q30: Each week, I do on average:
- At least 2 and a half hours of moderate intensity activity (such as brisk walking or cycling)
- Or at least 75 minutes of vigorous intensity activity (such as running)

- [1] YES
- [2] NO

Page 43

[SIMPLE_GRID][RADIO] Q30bis: Please indicate your level of agreement with this statement

Settings:

Mobile smart: **Yes**

Rows:

- [1] Physical activity and exercise may reduce the risk of future memory decline and dementia

Columns:

- [1] Strongly agree
- [2] Agree
- [3] Disagree
- [4] Strongly disagree
- [5] Don't know

Page 44

[SIMPLE_GRID][RADIO] Q40: In this section, we want to understand potential barriers for you to complete regular exercise

Settings:

Skip logic: **Q30 = 1**

Mobile smart: **Yes**

Instructions: Please rate your level of agreement with each statement

Rows:

- [1] I do not exercise regularly as I do not have access to facilities or guidance on exercise
- [2] I do not exercise regularly as it is too expensive to join a gym or fitness centre

Columns:

- [1] Strongly agree
- [2] Agree
- [3] Disagree
- [4] Strongly disagree

Page 45

[SIMPLE_GRID][RADIO] Q40_CLONE: In this section, we want to understand what are the potential barriers for you to complete regular exercise

Settings:

Skip logic: **Q30 = 1**

Mobile smart: **Yes**

Instructions: Please rate your level of agreement with each statement

Rows:

- [3] I do not exercise regularly as I have a physical disability
- [1] I want to exercise more but I do not have the drive to do it regularly

Columns:

- [1] Strongly agree
- [2] Agree
- [3] Disagree
- [4] Strongly disagree

Page 46

[SIMPLE_GRID][RADIO] Q31

Settings:

Skip logic: **Q30 = 1**

Show logic: **0**

Mobile smart: **Yes**

Instructions: Please rate your level of agreement with each statement

Rows:

- [2] Work commitments mean I do not have enough time to exercise

- [3] Home commitments mean I do not have enough time to exercise

Columns:

- [1] Strongly agree
- [2] Agree
- [3] Disagree
- [4] Strongly disagree

Page 47

[SIMPLE_GRID][RADIO] Q31_CLONE

Settings:

Skip logic: **0**

Show logic: **0**

Mobile smart: **Yes**

Instructions: Please rate your level of agreement with each statement

Rows:

- [1] Moderate exercise can have a positive impact on the mood
- [2] Moderate exercise can have a positive impact on the quality of sleep

Columns:

- [1] Strongly agree
- [2] Agree
- [3] Disagree
- [4] Strongly disagree
- [5] Don't know

Page 48

[CHECKBOX] Q32: The following types of exercise would be most appealing to me (tick all that apply)

Settings:

Skip logic: **(Q30 == 1)**

Min Count: **1**

- [1] Gentle walk
- [2] Brisk walk
- [3] Running
- [4] Cycling
- [5] Yoga
- [6] Swimming
- [7] None of the above / Other [DONTKNOW]

Page 49 - Logic : Logic blocks straight line

LOOP ALWAYS

Initialise vars to zero to start

For each response in **SL_Q31** as X:

Page 50

[RADIO] Q33: I currently have low mood or depression

- [1] YES
- [2] NO

[SIMPLE_GRID][RADIO] Q59: Please indicate your level of agreement with this statement

Settings:

Mobile smart: **Yes**

Rows:

- [1] Mood difficulties like depression may contribute to future risk of memory decline and dementia

Columns:

- [1] Strongly agree
- [2] Agree
- [3] Disagree
- [4] Strongly disagree
- [5] Don't know

Page 51

[SIMPLE_GRID][RADIO] Q34: In this section, we want to understand your attitudes to low mood and depression

Settings:

Enabled: **No**

Skip logic: **Q33 = 1**

Mobile smart: **Yes**

Instructions: Please rate your level of agreement with each statement

Rows:

- [1] If I had low mood or depression, I would know how to access services to help
- [2] If I had low mood or depression, I would be worried about asking others for help because of what they might think

Columns:

- [1] Strongly agree
- [2] Agree
- [3] Disagree
- [4] Strongly disagree

Page 52

[SIMPLE_GRID][RADIO] Q34_CLONE_CLONE: In this section, we want to understand your attitudes to low mood and depression

Settings:

Enabled: **No**

Skip logic: **Q33 = 1**

Mobile smart: **Yes**

Instructions: Please rate your level of agreement with each statement

Rows:

- [1] If I had low mood or depression, I would not seek help from healthcare professionals due to previous bad experiences or lack of trust in their services
- [2] If I had low mood or depression, this would mainly relate to life at home

Columns:

- [1] Strongly agree
- [2] Agree
- [3] Disagree
- [4] Strongly disagree

Page 53

[SIMPLE_GRID][RADIO] Q34_CLONE

Settings:

Enabled: **No**

Skip logic: **Q33 = 1**

Mobile smart: **Yes**

Instructions: Please rate your level of agreement with each statement

Rows:

- [2] If I had low mood or depression, this would mainly relate to work stress
- [3] If I had low mood or depression, this would mainly relate to financial worries

Columns:

- [1] Strongly agree
- [2] Agree
- [3] Disagree
- [4] Strongly disagree

Page 54

[SIMPLE_GRID][RADIO] Q34_CLONE_CLONE_CLONE

Settings:

Enabled: **No**

Skip logic: **Q33 = 1**

Mobile smart: **Yes**

Instructions: Please rate your level of agreement with this statement

Rows:

- [4] If I had low mood or depression, this would mainly relate to worries about my health

Columns:

- [1] Strongly agree
- [2] Agree
- [3] Disagree
- [4] Strongly disagree

Page 55

[SIMPLE_GRID][RADIO] Q34_CLONE2: In this section, we want to understand your attitudes to low mood and depression

Settings:

Skip logic: **Q33 = 2**

Mobile smart: **Yes**

Instructions: Please rate your level of agreement with each statement

Rows:

- [1] I know how to access services to help with low mood or depression
- [2] I am worried about asking others for help because of what they may think
- [3] I do not seek help from healthcare professionals due to previous bad experiences or lack of trust in their services

Columns:

- [1] Strongly agree
- [2] Agree
- [3] Disagree
- [4] Strongly disagree

Page 56

[SIMPLE_GRID][RADIO] Q34_CLONE3

Settings:

Skip logic: **Q33 = 2**

Mobile smart: **Yes**

Instructions: Please rate your level of agreement with each statement

Rows:

- [1] My low mood or depression mainly relates to life at home
- [2] My low mood or depression mainly relates to work related stress

Columns:

- [1] Strongly agree
- [2] Agree
- [3] Disagree

- [4] Strongly disagree

Page 57

[SIMPLE_GRID][RADIO] Q34_CLONE3_CLONE

Settings:

Skip logic: **Q33 = 2**

Mobile smart: **Yes**

Instructions: Please rate your level of agreement with each statement

Rows:

- [3] My low mood or depression mainly relates to financial worries
- [4] My low mood or depression mainly relates to worries about my health

Columns:

- [1] Strongly agree
- [2] Agree
- [3] Disagree
- [4] Strongly disagree

Page 58 - Logic : Logic blocks straight line

LOOP ALWAYS

Initialise vars to zero to start

For each response in **SL_Q34_CLONE** as X:

Page 59

[RADIO] Q35: I have diabetes (Type I or II, diet or medication controlled)

- [1] YES
- [2] NO

[SIMPLE_GRID][RADIO] Q51: Please indicate your level of agreement with this statement

Settings:

Mobile smart: **Yes**

Rows:

- [1] Having good blood sugar control may reduce the risk of future memory decline and dementia

Columns:

- [1] Strongly agree
- [2] Agree
- [3] Disagree
- [4] Strongly disagree
- [5] Don't know

Page 60

[SIMPLE_GRID][RADIO] Q36: In this section, we want to understand the barriers you may have to adequately manage your diabetes

Settings:

Skip logic: **Q35 = 2**

Mobile smart: **Yes**

Instructions: Please rate your level of agreement with each statement

Rows:

- [1] My sugar levels are normally very well controlled
- [2] I know how to seek assessment and advice for diabetes

Columns:

- [1] Strongly agree
- [2] Agree
- [3] Disagree
- [4] Strongly disagree

Page 61

[SIMPLE_GRID][RADIO] Q36_CLONE_CLONE: In this section, we want to understand the barriers you may have to adequately manage your diabetes

Settings:

Skip logic: **Q35 = 2**

Mobile smart: **Yes**

Instructions: Please rate your level of agreement with this statement

Rows:

- [3] I often forget to take my diabetes medication at the correct time

Columns:

- [1] Strongly agree
- [2] Agree
- [3] Disagree
- [4] Strongly disagree

Page 62

[SIMPLE_GRID][RADIO] Q36_CLONE: In this section, we want to understand the barriers you may have to adequately manage your diabetes

Settings:

Skip logic: **Q35 = 2**

Mobile smart: **Yes**

Instructions: Please rate your level of agreement with each statement

Rows:

- [1] I do not have the time to monitor my sugar levels as often as I should
- [2] I am too worried to get my sugar levels checked in case a problem is found

Columns:

- [1] Strongly agree
- [2] Agree
- [3] Disagree
- [4] Strongly disagree

Page 63 - Logic : Logic blocks straight line

LOOP ALWAYS

Initialise vars to zero to start

For each response in **SL_Q36_CLONE** as X:

Page 64

[SIMPLE_GRID][RADIO] Q59_CLONE: Please indicate your level of agreement with this statement **regarding social interactions**

Settings:

Mobile smart: **Yes**

Rows:

- [1] Increased social interactions may reduce the risk of future memory decline and dementia

Columns:

- [1] Strongly agree
- [2] Agree
- [3] Disagree

- [4] Strongly disagree
- [5] Don't know

Page 65

[SIMPLE_GRID][RADIO] Q37: In this section, we want to understand potential barriers you may have to social engagement and interactions

Settings:

Mobile smart: **Yes**

Instructions: Please rate your level of agreement with each statement

Rows:

- [1] I do not socialise much, as I like being by myself and enjoy my own company
- [2] I do not have access to facilities like social clubs or social events to meet and see other people

Columns:

- [1] Strongly agree
- [2] Agree
- [3] Disagree
- [4] Strongly disagree

Page 66

[SIMPLE_GRID][RADIO] Q37_CLONE_CLONE: In this section, we want to understand the barriers you may have to social engagement and interaction

Settings:

Mobile smart: **Yes**

Instructions: Please rate your level of agreement with each statement

Rows:

- [3] I do not have many friends or family to socialise with
- [4] I don't have enough time to socialise regularly with friends or family

Columns:

- [1] Strongly agree
- [2] Agree
- [3] Disagree
- [4] Strongly disagree

Page 67

[SIMPLE_GRID][RADIO] Q37_CLONE

Settings:

Mobile smart: **Yes**

Instructions: Please rate your level of agreement with each statement

Rows:

- [1] I live too far away from friends or family to socialise with them on a regular basis
- [2] I do not socialise often as I have stress / anxiety around meeting others

Columns:

- [1] Strongly agree
- [2] Agree
- [3] Disagree
- [4] Strongly disagree

Page 68

[SIMPLE_GRID][RADIO] Q37_CLONE_CLONE_CLONE

Settings:

Mobile smart: **Yes**

Instructions: Please rate your level of agreement with each statement

Rows:

- [3] I do not socialise with friends or family because I cannot afford the costs of going out and meeting people
- [4] I do not socialise as much anymore because I am worried about COVID-19

Columns:

- [1] Strongly agree
- [2] Agree
- [3] Disagree
- [4] Strongly disagree

Page 69 - Logic : Logic blocks straight line

LOOP ALWAYS

Initialise vars to zero to start

For each response in **SL_Q37_CLONE** as X:

Page 70

[RADIO] Q38: I drink alcohol (even if occasionally)

- [1] YES
- [2] NO

[SIMPLE_GRID][RADIO] Q52: Please indicate your level of agreement with this statement

Settings:

Skip logic: **0**

Mobile smart: **Yes**

Rows:

- [1] Excessive consumption of alcohol may increase the risk of future memory decline and dementia

Columns:

- [1] Strongly agree
- [2] Agree
- [3] Disagree
- [4] Strongly disagree
- [5] Don't know

Page 71

[RADIO] Q39_CLONE_2: On average, I drink more than 14 units of alcohol per week (1 unit is the equivalent of half a pint of beer or a small glass of wine)

Settings:

Skip logic: **(Q38 == 2)**

- [1] YES
- [2] NO

Page 72

[SIMPLE_GRID][RADIO] Q39: In this section, we want to understand your attitudes to alcohol consumption

Settings:

Skip logic: **Q38 = 2**

Show logic: **0**

Mobile smart: **Yes**

Instructions: Please rate your level of agreement with each statement

Rows:

- [1] I don't want to reduce my alcohol intake as I do not feel there is a need
- [2] I don't think I can reduce my alcohol intake due to stress from work or personal reasons and I feel alcohol helps me deal with this

Columns:

- [1] Strongly agree
- [2] Agree
- [3] Disagree
- [4] Strongly disagree

Page 73

[SIMPLE_GRID][RADIO] Q39_CLONE: In this section, we want to understand your attitudes to alcohol consumption

Settings:

Skip logic: **Q38 = 2**

Show logic: **0**

Mobile smart: **Yes**

Instructions: Please rate your level of agreement with each statement

Rows:

- [3] I want to reduce my alcohol intake, however, I don't feel I can reduce it when the people I live with continue to drink alcohol
- [4] I don't feel I can reduce my alcohol intake due to the social life I have

Columns:

- [1] Strongly agree
- [2] Agree
- [3] Disagree
- [4] Strongly disagree

Page 74 - Logic : Logic blocks straight line

LOOP ALWAYS

Initialise vars to zero to start

For each response in **SL_Q39** as X:

Page 75

[RADIO] Q41: I take part in activities that increase the risk of head injury, such as boxing or rugby

- [1] YES
- [2] NO

[SIMPLE_GRID][RADIO] Q53: Please indicate your level of agreement with this statement

Settings:

Mobile smart: **Yes**

Rows:

- [1] Significant or repetitive head injuries may increase the risk of future memory decline and dementia

Columns:

- [1] Strongly agree
- [2] Agree
- [3] Disagree
- [4] Strongly disagree
- [5] Don't know

Page 76

[SIMPLE_GRID][RADIO] Q42: In this section, we want to understand the barriers you may have to reducing the risk of head injury

Settings:

Skip logic: **Q41 = 2**

Show logic: **0**

Mobile smart: **Yes**

Instructions: Please rate your level of agreement with each statement

Rows:

- [1] I enjoy taking part in contact sports or other activities too much to stop doing them
- [2] Activities that increase the risk of head injury are part of my life

Columns:

- [1] Strongly agree
- [2] Agree
- [3] Disagree
- [4] Strongly disagree

Page 77

[SIMPLE_GRID][RADIO] Q43: Please indicate your level of agreement with this statement **regarding air pollution**

Settings:

Mobile smart: **Yes**

Rows:

- [1] Prolonged exposure to poor air quality and pollution may increase the risk of future memory decline and dementia

Columns:

- [1] Strongly agree

- [2] Agree
- [3] Disagree
- [4] Strongly disagree
- [5] Don't know

Page 78

[SIMPLE_GRID][RADIO] Q44: Please rate your level of agreement with each statement

Settings:

Skip logic: **Q43 = 2**

Show logic: **0**

Mobile smart: **Yes**

Rows:

- [1] I live or work in an area with a relatively high level of air pollution
- [2] If I wanted to, I could afford to change the area I live or work in to reduce the amount of air pollution I am exposed to

Columns:

- [1] Strongly agree
- [2] Agree
- [3] Disagree
- [4] Strongly disagree

Page 79

[RADIO] Q45: Generally, I am able to get a good night's sleep every night.

- [1] YES
- [2] NO

[SIMPLE_GRID][RADIO] Q54: Please indicate your level of agreement with this statement

Settings:

Mobile smart: **Yes**

Rows:

- [1] Poor sleep may increase the risk of future memory decline and dementia

Columns:

- [1] Strongly agree
- [2] Agree
- [3] Disagree
- [4] Strongly disagree
- [5] Don't know

Page 80

[SIMPLE_GRID][RADIO] Q46_CLONE: In this section, we want to understand the barriers you may have to getting a good night's sleep

Settings:

Skip logic: **Q45 = 1**

Show logic: **0**

Mobile smart: **Yes**

Instructions: Please rate your level of agreement with each statement

Rows:

- [1] I don't often get a good night's sleep because I am too stressed or find it difficult to unwind
- [2] I don't often get a good night's sleep because of family commitments

Columns:

- [1] Strongly agree

- [2] Agree
- [3] Disagree
- [4] Strongly disagree

Page 81

[SIMPLE_GRID][RADIO] Q46_CLONE_CLONE: In this section, we want to understand the barriers you may have to getting a good night's sleep

Settings:

Skip logic: **Q45 = 1**

Show logic: **0**

Mobile smart: **Yes**

Instructions: Please rate your level of agreement with this statement

Rows:

- [3] I don't often get a good night's sleep because of work commitments

Columns:

- [1] Strongly agree
- [2] Agree
- [3] Disagree
- [4] Strongly disagree

Page 82

[SIMPLE_GRID][RADIO] Q46

Settings:

Skip logic: **Q45 = 1**

Show logic: **0**

Mobile smart: **Yes**

Instructions: Please rate your level of agreement with each statement

Rows:

- [1] I don't often get a good night's sleep because I live in a noisy area
- [2] I don't often get a good night's sleep because of my caffeine intake

Columns:

- [1] Strongly agree
- [2] Agree
- [3] Disagree
- [4] Strongly disagree

Page 83

[SIMPLE_GRID][RADIO] Q46_CLONE_CLONE_CLONE

Settings:

Skip logic: **Q45 = 1**

Show logic: **0**

Mobile smart: **Yes**

Instructions: Please rate your level of agreement with this statement

Rows:

- [3] I don't often get a good night's sleep and I don't know why

Columns:

- [1] Strongly agree
- [2] Agree
- [3] Disagree
- [4] Strongly disagree

Page 84 - Logic : Logic blocks straight line

LOOP ALWAYS

Initialise vars to zero to start

For each response in **SL_Q46** as X:

Page 85

[RADIO] Q47: I often take part in **mentally stimulating activities**, such as reading, crossword puzzles, chess, learning a new language or playing an instrument

- [1] YES
- [2] NO

[SIMPLE_GRID][RADIO] Q55: Please indicate your level of agreement with this statement

Settings:

Mobile smart: **Yes**

Rows:

- [1] A lack of engagement in mentally stimulating activities may increase the risk of memory decline and dementia

Columns:

- [1] Strongly agree
- [2] Agree
- [3] Disagree
- [4] Strongly disagree
- [5] Don't know

Page 86

[SIMPLE_GRID][RADIO] Q48: In this section, we want to understand the barriers you may have to engage in mentally stimulating activities

Settings:

Skip logic: **Q47 = 1**

Mobile smart: **Yes**

Instructions: Please rate your level of agreement with each statement

Rows:

- [1] I don't often have the time to engage in mentally stimulating activities
- [2] I don't find mentally stimulating activities entertaining

Columns:

- [1] Strongly agree
- [2] Agree
- [3] Disagree
- [4] Strongly disagree

Page 87

[SIMPLE_GRID][RADIO] Q48_CLONE: In this section, we want to understand the barriers you may have to engage in mentally stimulating activities

Settings:

Skip logic: **Q47 = 1**

Mobile smart: **Yes**

Instructions: Please rate your level of agreement with each statement

Rows:

- [3] I don't engage in mentally stimulating activities as I do not have the financial means to do so
- [4] I don't take part in mentally stimulating activities because I don't have any close family members or friends to do them with

Columns:

- [1] Strongly agree
- [2] Agree
- [3] Disagree
- [4] Strongly disagree
